# False-Positive ^18^F-FDG PET/CT Uptake in Unilateral Lactation

**DOI:** 10.1155/2020/8850052

**Published:** 2020-11-28

**Authors:** Joshua Wei Liang Yip, Han Loh, Chuong Bui, Veronica Chi Ken Wong, Robert Mansberg

**Affiliations:** ^1^Department of Nuclear Medicine and PET, Nepean Hospital, Derby St, Kingswood NSW 2747, Australia; ^2^School of Medicine, University of Sydney, NSW, Australia

## Abstract

A 31-year-old woman (7 months postpartum and lactating) with multiple sclerotic bone lesions was referred for an ^18^F-FDG PET/CT scan for characterization. The scan demonstrated unilateral diffuse intense FDG uptake corresponding to dense soft tissue in the right breast, likely related to secretory hyperplasia. On further questioning, it was made apparent that she had only been breastfeeding from the right breast. While the left breast also demonstrated dense soft tissue to a lesser degree, no significant FDG uptake was seen. The sclerotic bone lesions were not FDG avid, likely due to a separate non-FDG avid benign condition or bony metastases from a non-FDG avid primary malignancy. This was reinforced by the fact that subsequent investigations including serial bilateral breast ultrasound and percutaneous biopsy demonstrated no definite evidence of malignancy in the bilateral breasts. The histopathology findings of an open surgical biopsy of sclerotic lesions in the left posterior ilium were also nonspecific, favouring bone dysplasia with no evidence of malignancy.

## 1. Case Presentation

**Figure 1:**
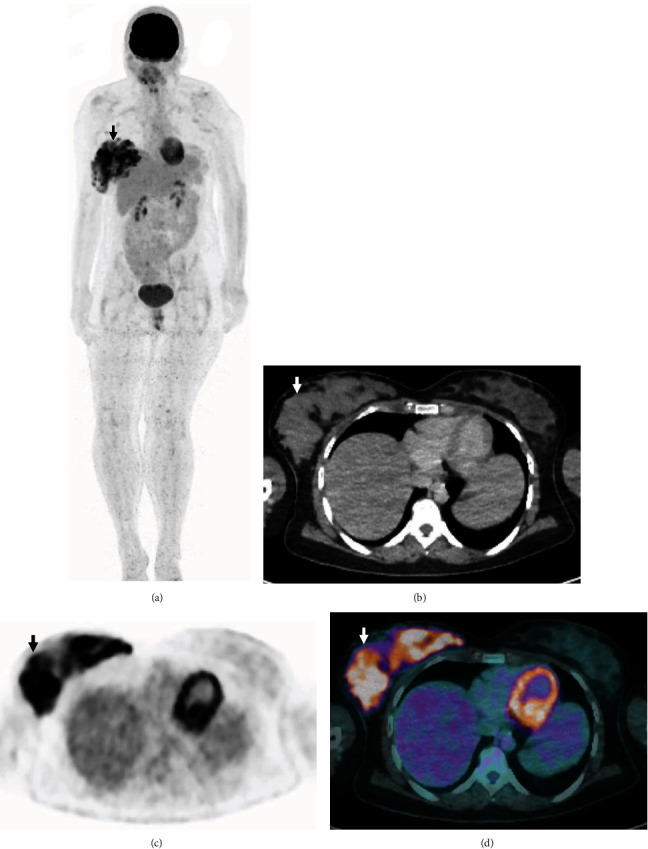
The maximum intensity projection (a) image demonstrates unilateral diffuse intense FDG uptake in the right breast (arrow). Axial low dose CT (b), FDG PET (c), and fused FDG PET/CT (d) images demonstrate unilateral diffuse intense FDG uptake (SUVmax up to 12.8) corresponding to dense soft tissue in the right breast (arrows).

**Figure 2:**
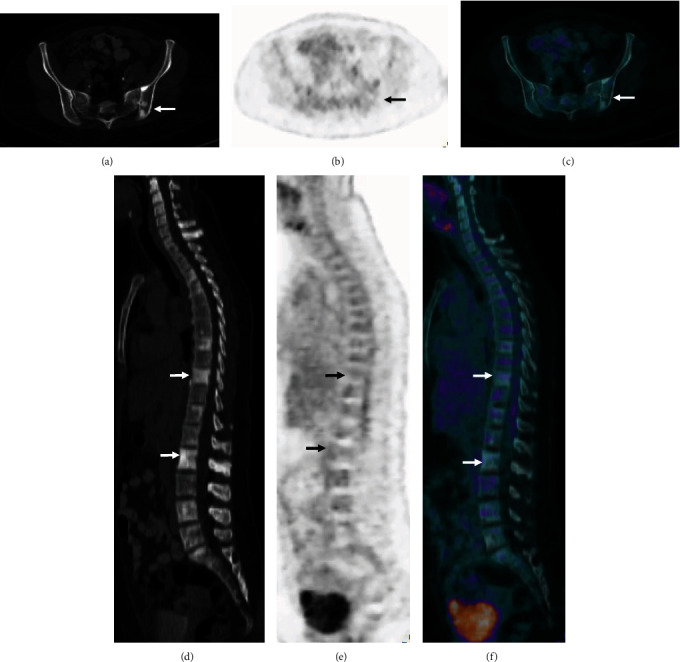
Axial low dose CT (a), FDG PET (b), and fused FDG PET/CT (c) images demonstrate sclerotic bone lesions in the bony pelvis with no significant FDG uptake (arrows). Sagittal CT (d), FDG PET (e), and fused FDG PET/CT (f) images demonstrate sclerotic bone lesions throughout the spine with no significant FDG uptake (arrows).

A 31-year-old lactating woman was referred for investigation of multiple sclerotic bone lesions, 7 months since inception of breastfeeding. Following appropriate radiation safety advice where the patient was instructed to discard expressed breast milk for 4 hours, the patient was injected with 5.4 mCi (200 MBq) ^18^F-FDG and imaged on a GE Discovery PET/CT camera (Figures 1 and 2).

The unilateral diffuse intense FDG uptake corresponding to dense soft tissue in the right breast was likely due to secretory hyperplasia as further questioning confirmed that the patient had been breastfeeding only with the right breast due to her infant's preferences, as the infant was refusing to feed from the contralateral left breast. No significant FDG uptake was seen in the contralateral left breast which was not used for breastfeeding, 7 months after childbirth, although it also demonstrated dense soft tissue to a lesser degree. The sclerotic bone lesions were not FDG avid, likely due to a separate non-FDG avid benign condition or bony metastases from a non-FDG avid primary malignancy.

Extensive investigations were carried out to rule out the possibility of metastatic breast cancer. Serial bilateral breast ultrasound at the time of the PET study and 5 months later demonstrated stable bilateral similar well-defined hypoechoic lesions in the right breast measuring 15 mm and in the left breast measuring 21 mm, favouring benign aetiologies such as fibroadenomas or galactoceles. Percutaneous biopsy of the larger left breast lesion confirmed a fibroadenoma. In discussion with the patient, the second smaller lesion with similar appearances in the right breast was not biopsied in light of its stable benign appearance and in keeping with the patient's preferences. Percutaneous bone biopsy of a sclerotic lesion in the left iliac bone was nondiagnostic due to bone dust contamination. The patient subsequently underwent an open surgical biopsy of the left posterior ilium where visible sclerotic lesions were evident on gross examination during the surgery. The histopathology findings of this surgical biopsy were nonspecific, but favoured bone dysplasia with no malignant cells seen.

## 2. Discussion

In pregnancy and lactation, the major physiological changes in the breast are due to variations in the serum estrogen, progesterone, and prolactin. During pregnancy, elevated estrogen and progesterone levels are responsible for ductal and lobular hyperplasia and enable the alveolar cells to secrete milk. Prolactin, which plays a role in stimulating the production of milk, incrementally increases during pregnancy but drops to baseline levels after childbirth. Lactation then is sustained by continued breastfeeding, which causes a biofeedback loop resulting in further prolactin secretion [1]. In response to these hormones, changes to the breast include marked ductular and lobular growth, increased glandular vascularity, and fibrofatty stromal involution [2]. As a result of which, lactating breasts demonstrate enlargement with bilateral high attenuation tissue [3]. Glucose is utilised as a fuel and as a component of milk, playing a large role in mammary gland function in lactation [3]. It is known that lactating breasts used for feeding have been shown to demonstrate increased FDG uptake, and case studies exist to suggest that unused lactating breasts do not demonstrate significant FDG uptake [4–7]. This suggests that the intense FDG uptake demonstrated in lactating breasts is likely due to suckling rather than prolactin, which is postulated to increase expression of glucose transporter-1 (GLUT-1) in epithelial cells [3, 5, 8]. Other differentials for unilateral diffuse FDG uptake in the breast include advanced breast carcinoma, lymphoma, and inflammation [5, 9, 10]. While more sinister pathology should be excluded, physicians should be aware of the importance of clinical history in patients who are breastfeeding that demonstrate diffuse FDG uptake in the breast, which could be due to secretory hyperplasia and the increased expression of GLUT-1. Atypical findings such as unilateral breast FDG uptake should be further clarified with the patient to avoid false-positive diagnosis.
